# Outlying Sanatoria

**Published:** 1920-05-29

**Authors:** 


### Outlying Sanatoria.

A question was lately asked in Parliament as to the action of the Dorsetshire authorities in using a house in the centre of Wiltshire as a tuberculosis sanatorium. This action some Wiltshire people, being not apparently of the mind of a celebrated novelist in considering the whole of South-West England as one province, seemed to resent. Dr. Addison, in reply, let it be known that the property had been presented to Dorset, and as it was suitable to the purpose contemplated, and would be productive of no detriment, would be so used. He added that if the Ministry were to take heed to all the objections raised against sanatorium sites, there would be none of these institutions at all. This is undoubtedly the case, but the Minister might have added that such objections are usually very short-lived, being replaced by a sense of financial advantage to the neighbourhood. In the case under notice the Dorsetshire tradesmen's loss will be the Wiltshire tradesmen's gain; that and the difficulty of access for patients and their visitors constitute the only real objections.

